# Allelic Variant in the Anti-Müllerian Hormone Gene Leads to Autosomal and Temperature-Dependent Sex Reversal in a Selected Nile Tilapia Line

**DOI:** 10.1371/journal.pone.0104795

**Published:** 2014-08-26

**Authors:** Stephan Wessels, Reza Ahmad Sharifi, Liane Magdalena Luehmann, Sawichaya Rueangsri, Ina Krause, Sabrina Pach, Gabriele Hoerstgen-Schwark, Christoph Knorr

**Affiliations:** 1 Department of Animal Sciences - Aquaculture and Water Ecology, Goettingen University, Goettingen, Germany; 2 Department of Animal Sciences - Animal Breeding and Genetics, Goettingen University, Goettingen, Germany; 3 Department of Animal and Aquatic Sciences, Faculty of Agriculture, Chiang Mai University, Chiang Mai, Thailand; 4 Department of Animal Sciences - Molecular Biology and Molecular Diagnostics of Livestock, Goettingen University, Goettingen, Germany; 5 Department of Animal Sciences - Livestock Biotechnology and Reproduction, Goettingen University, Goettingen, Germany; Temasek Life Sciences Laboratory, Singapore

## Abstract

Owing to the demand for sustainable sex-control protocols in aquaculture, research in tilapia sex determination is gaining momentum. The mutual influence of environmental and genetic factors hampers disentangling the complex sex determination mechanism in Nile tilapia (*Oreochromis niloticus*). Previous linkage analyses have demonstrated quantitative trait loci for the phenotypic sex on linkage groups 1, 3, and 23. Quantitative trait loci for temperature-dependent sex reversal similarly reside on linkage group 23. The anti-Müllerian hormone gene (*amh*), located in this genomic region, is important for sexual fate in higher vertebrates, and shows sexually dimorphic expression in Nile tilapia. Therefore this study aimed at detecting allelic variants and marker-sex associations in the *amh* gene. Sequencing identified six allelic variants. A significant effect on the phenotypic sex for SNP *ss831884014* (p<0.0017) was found by stepwise logistic regression. The remaining variants were not significantly associated. Functional annotation of SNP *ss831884014* revealed a non-synonymous amino acid substitution in the *amh* protein. Consequently, a fluorescence resonance energy transfer (FRET) based genotyping assay was developed and validated with a representative sample of fish. A logistic linear model confirmed a highly significant effect of the treatment and genotype on the phenotypic sex, but not for the interaction term (treatment: p<0.0001; genotype: p<0.0025). An additive genetic model proved a linear allele substitution effect of 12% in individuals from controls and groups treated at high temperature, respectively. Moreover, the effect of the genotype on the male proportion was significantly higher in groups treated at high temperature, giving 31% more males on average of the three genotypes. In addition, the groups treated at high temperature showed a positive dominance deviation (+11.4% males). In summary, marker-assisted selection for *amh* variant *ss831884014* seems to be highly beneficial to increase the male proportion in Nile tilapia, especially when applying temperature-induced sex reversal.

## Introduction

Tilapias, famed for their rapid and efficient growth, their low position on the food chain and deemed the “aquatic chicken”, provide a possibility to nourish the poor and to conquer export markets. Even though intensive aquaculture farming resulted in rapid breeding and stunted populations, hybridisation or application of synthetic hormones (17α-methyl testosterone) allowed the production of mono-sex male broods [1]. The latter technique can effectively prevent fry production before harvest. Showcasing tilapia as the first aquaculture fish species certified by the Aquaculture Stewardship Council [2], this emphasizes the need for more sustainable sex control protocols. As such, a temperature treatment of the fry might be a future alternative [3], [4]. The complex sex determination (SD) in Nile tilapia is comprised of an interaction between genetic (major and minor factors) and environment-/temperature-dependent factors [5]. Due to its complex SD, Nile tilapia is a well-suited model species for the evolution of genetic or environmental sex-determining mechanisms. Although an increasing number of genomic tools has been developed for Nile tilapia, such as a genetic map with formerly 23 linkage groups (LG) [6], which has recently been resolved in 22 LGs in a high-density radiation hybrid map with 1358 genetic markers [7], BAC end sequences [8], and an assembled draft of its genome (http://www.broadinstitute.org/scientific-community/science/projects/mammals-models/vertebrates-invertebrates/tilapia-/tilapia-genom), a comprehensive understanding of the SD mechanism is still outstanding. On the one hand, linkage analysis demonstrated some success, with QTL found for phenotypic sex on LG 1, 3, and 23 in intraspecific and interspecific crosses of tilapia species [9]–[17]. On the other hand, neither assigning vertebrate SD candidate genes to tilapia linkage groups [18]–[20] nor the analysis of expressed sequence tags for ovary- and testis-specific libraries resulted in appreciable success so far [21]. Beside major QTL on LG1 and LG3 [22], Eshel et al. 2012 recently fine-mapped a SD region on LG23 [15]. The QTL mapped to 13–40 cM and more precisely peaked at 22 cM (F = 78.7; P<7.6×10^−14^). Linkage mapping located the SD QTL between markers *GM597* and *ARO124*. The QTL region, located in scaffold 101 of the Nile tilapia genome assembly, harbours the anti-Müllerian hormone gene (*amh*), among others. A further study proposed that temperature-dependent sex reversal similarly seems to be influenced in at least some families by an even broader QTL region (between *GM283* and *UNH898*) on LG23 [23].

The *amh* gene is involved in the development of the urogenital system during embryogenesis by suppressing the Müllerian ducts of mammals, birds and reptiles [24]–[26]. Both *amh* and its target in the *TGF-ß* pathway, the type II *amh* receptor (*amhrII*), are expressed in Sertoli cells of teleost [27]. Moreover, *amh* has been cloned in a number of fish species despite the fact that they lack Müllerian ducts [28]–[31]. The expression of the *amh* gene can be detected starting at 3 days post fertilisation (dpf) in XX and XY gonads [15]. The authors also demonstrated a significantly higher expression of *amh* in all-male vs. all-female batches at 3 dpf. Differences became more pronounced until 7 dpf [15]. Earlier investigations tended to show dimorphic expression in the gonads at later stages, i.e. after *dmrt1* at 19 dpf [32]. Moreover, sustained up regulation of *dmrt1* and *amh* during temperature sex-reversal from 13–15 dpf onwards and down regulation of *foxl2* and/or *cyp19a1a* at 17–19 dpf were observed during sexual development of Nile tilapia [33]. Even in the brain, *amh* expression appeared to be clearly sexually dimorphic during the period from 10 to 15 dpf [34].

This study reports the detection of allelic variants in the coding and 5′ upstream region of *amh*. The development of a high-throughput genotyping assay for a single SNP and its association with the phenotypic sex let *amh* appear as a candidate for autosomal and temperature-induced sexual development in Nile tilapia.

## Materials and Methods

### Stocks and Cross Design

**Figure 1:**
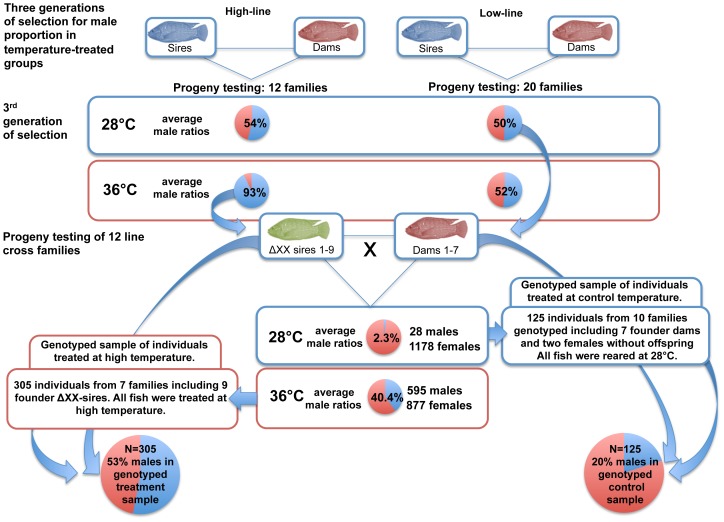
Experimental design to obtain a genetically female (XX) population in order to study temperature effects on the male proportion in a selected line of Nile tilapia.

The aim of the present study was to identify alleles in the *amh* gene with an influence on autosomal and temperature-dependent sex reversal. For this purpose genetically all-female (XX) populations were used, which had been tested for their chromosomal sex (XX vs. XY) via progeny testing (Figure 1). The genetically all-female population was derived from crosses between females from a selected line for low responsiveness to temperature (<60% males after 36°C treatment from 10–20 dpf) and temperature-sex reversed males from a line for high temperature-responsiveness (>90% males after 36°C treatment from 10–20 dpf) [3], [4]. The genetically female line was developed through mating of 9 sex-reversed (ΔXX) sires derived from 7 high-line families, which were treated at high temperature, to 7 females from 6 low-line families in order to produce a total of twelve families. One line cross family was derived from a within line mating (sire 105, dam 32, Table S1 in File S1). Briefly, progenies were obtained through artificial reproduction (Figure 1). Fertilised eggs were incubated for 10 days at 28°C. After yolk sac absorption, larvae from each family were randomly distributed into two groups (n∼110 larvae each). Temperature in the control groups was 28°C throughout the experiment, whereas treatment groups were kept at 36±0.5°C from 10–20 dpf according to [3]. From 20 dpf onwards, control and groups treated at high temperature were raised in separate tanks at 28°C for at least 2 months. From each family treated at high temperature 15 adult males and 15 adult females were phenotyped alive, assessing the urogenital papilla and the types of gametes each fish produced. All individuals that could not be confidently phenotyped, even after repeated inspection of the genital papilla, were excluded from further analysis. The remaining fish from the groups treated at high temperature and control groups were subjected to a lethal dose of phenoxyethanol (ethylene glycol monophenyl ether at 500 µL/L) and were immediately exanguated and dissected. The latter fish were phenotyped for their sex based on microscopical inspection of squashed gonads according to [35], classifying them into either testes or ovaries.

Tissue samples from all individuals were collected from caudal fins and stored at −20°C until DNA extraction. The DNA was isolated from a sample of finclips by phenol-chloroform extraction [36]. The sample was comprised of 305 individuals from 7 out 12 line cross families including all 9 ΔXX founder sires of the 12 line cross families, which were all treated at high temperature. Furthermore DNA was extracted from 125 control individuals from 10 out of 12 line cross families including the 7 founder dams of the 12 line cross families and two additional females with no offspring. The numbers of fish genotyped from each family and corresponding control or treatment group are given in Table S1 and S5 in File S1. Water parameters measured during the experimental period were within the following range: oxygen >5.5 mg/L; pH 6.5–7.5; NH^4+^<0.5 mg/L; NO^2−^<0.25 mg/L. For first feeding, the fish were provided a diet rich in protein three times a day ad libitum (Tetra Werke, Germany; crude protein 48.5%). Generally, from day 20 until day 60, fish were fed ad libitum with a trout feed (Skretting F0 Aqua Brut, Norway; crude protein 48.5%) while from day 60 the fish were provided a carp feed (Skretting C2 Pro Aqua K18, Norway; crude protein 36%). All procedures were in strict accordance with the recommendations in the Guide for the Care and Use of Laboratory Animals of the German Animal Welfare Act [37]. This study was approved by the Institutional Animal Care and Use Committee of Goettingen University.

### Sequencing of the *amh* gene

The genomic sequence of the *amh* gene was derived from Scaffold *GL831234.1* of the Nile tilapia genome sequence deposited in the Ensembl database (http://www.ensembl.org; *Orenil1.0 GCA_000188235.1*; location of *amh*: Scaffold *GL831234.1*, 1.688.687–1.691.779). Gene-specific primers were designed to cover the coding sequence (cds), including intron and 5′UTR regions of the *amh* gene using the Primer3 software (see Table S2 in File S1). Each forward or reverse primer for sequencing of the *amh* gene was tailed at the 5′end with the M13 universal forward or reverse primer to enable direct bidirectional sequencing on an 3130xL Genetic Analyzer (Applied Biosystems, Germany) using the BigDye Terminator v3.1 Cycle Sequencing Kit (Applied Biosystems). DNA for sequencing of the *amh* gene was derived from 93 individuals comprised three families including the corresponding three dams and sires (Table S6 in File S1). PCR was carried out using 20 ng of genomic DNA, 1× PCR buffer containing MgCl_2_, 1× Q-solution, 10 pmol of each primer, 10 mM dNTPs and 2 U FastStart Taq DNA polymerase, in a final volume of 25 µl. All PCR components except the primers (MWG, Germany) and the 1× Q-solution (Qiagen, Germany) were purchased from Roche Diagnostics (Mannheim, Germany). PCR was performed using a Biometra T-3000 Thermocycler (Goettingen, Germany) with an initial denaturation at 95°C for 10 min, followed by 35 cycles of 92°C for 30 s; 60°C for 30 s and 72°C for 1 min with a final extension at 72°C for 5 min. The fragment identity was controlled via gel-electrophoresis on 1.5–2% agarose gels. PCR products were then purified with Exo-SAP-IT (USB, Germany). The obtained sequences were trimmed, contigs were built, and SNPs were manually identified using the program software suite DNASTAR Lasergene6 (DNASTAR, Inc., Germany).

### Genotyping of SNP *ss831884014* within *amh*

**Figure 2:**
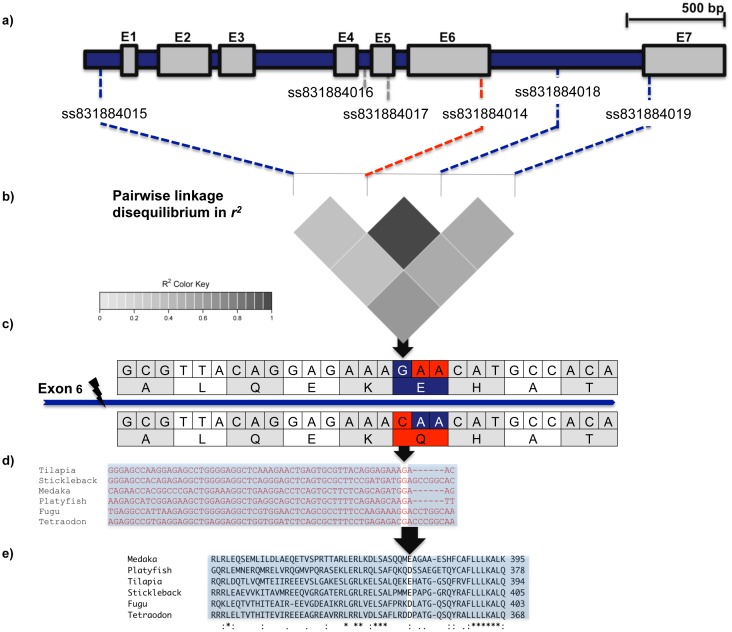
Gene structure, pairwise linkage disequilibrium (*r^2^*) between SNPs, and functional annotation of the Nile tilapia *amh* gene derived from the Ensembl database. a) Gene structure of the *amh* gene derived from Scaffold *GL831234.1*. Exons 1–7 (E1–7) are represented by filled grey boxes. Blue and red dashed lines show positions of polymorphic SNPs in the *amh* gene. SNP *ss831884015* (C>T) is located −225 bp upstream of the start codon ATG, SNP *ss831884018* was located in the intron between exon 6 and 7. Variants *ss831884014* and *ss831884019* were found in exons 6 and 7. SNP *ss831884014* (G>C) and *ss831884019* (C>T) were missense mutations leading to amino acid changes from glutamic acid to glutamine (codons Gaa/Caa) and alanine to valine (codons gCg/gTg). Table 1 deals with the functional annotation of all six SNPs. b) Pairwise linkage disequilibrium heat map of four polymorphic allelic variants in the Nile tilapia *amh* gene. c) Partial DNA to protein translation of exon 6 in the Nile tilapia *amh* gene, depicting a non-synonymous amino acid substitution at position 376 of the putative *amh*-protein (indicated by black arrow) coded by an allelic variant *ss831884014* of scaffold *GL831234.1*. d) Partial comparative alignment of the *amh* genomic exon 6 sequences for six teleost species derived from the Ensembl database. e) Partial comparative alignment of the *amh* protein sequence for six teleost species derived from the Ensembl database.

Comparative sequencing revealed six SNPs within the *amh* gene (Figure 2). To genotype SNP *ss831884014*, exhibiting the largest effect on the phenotypic sex, a fluorescence resonance energy transfer (FRET) was developed using a LightCycler 480 instrument (Roche Diagnostics, Mannheim, Germany). Primers Fret-for and Fret-rev were designed to flank SNP *ss831884014* yielding a fragment of 122 bp length. A 5′ Rox labelled fluorescent anchor probe (acceptor) was designed with a phosphorylated 3′ tail. The fluorescent sensor probe (donor) was designed with a 3′ Fam modification (Table S3 in File S1). At the 3′-end of the sensor probe the donor fluorescent molecule is excited at a wavelength of 533 nm. The fluorescent acceptor molecule at the 5′-end of the anchor probe receives the emitted energy from the donor. The emission of the fluorescence signal from the acceptor molecule is measured by the LightCycler 480 instrument using a filter combination of 483–610 nm. The sensor probe was designed to match the G-allele of SNP *ss831884014*. In the case of a mismatch due to presence of the C-allele, a lower melting temperature is detected. The LightCycler 480 software was adjusted to automatically detect genotypes based on melting curve results. All genotypes were manually checked for genotyping errors, deciphered in triplicates via Sanger sequencing. Homozygous C/C individuals showed a fluorescence peak between 483 and 610 nm wavelength at 58°C, whereas homozygous G/G individuals showed one at 64°C, and heterozygous fish showed two peaks (Figure S1 in File S1). The melting curve analysis comprised of an initial denaturation step (95°C for 1 min), a step rapidly lowering the temperature to 40°C and holding for 30 sec, and a heating step slowly increasing the temperature up to 80°C under a continuous measurement of fluorescence (1 acquisition/°C). In total 337 individuals were investigated using the FRET assay, in order to obtain a total of 430 genotypes at SNP *ss831884014* (including the before mentioned Sanger-sequenced individuals). Briefly, the PCR was run in a 25 µl reaction volume consisting of 1×PCR buffer containing MgCl_2_, 0.4 pmol of each primer and each FRET probe, 10 mM dNTPs, 2 U FastStart Taq DNA polymerase, H_2_O and 20 ng of DNA. All PCRs were carried out in 96 well plates in a LightCycler 480 with an initial denaturation at 95°C for 10 min, followed by 35 cycles of 92°C for 30 s, 56°C for 30 s and 72°C for 30 s.

### Statistical analysis

For the segregating SNPs (n = 4) the gene diversity, the allele- as well as genotype frequencies were analysed using the SAS/Genetics 9.3 software (SAS Inst., Inc., Cary, NC, USA). Phased parental haplotypes were reconstructed using the software package PLINK for 93 animals treated at high temperature [38]. The 93 individuals comprised three families including the corresponding three dams and sires. *R^2^*-values were calculated in SAS/Genetics 9.3 software as a measure of linkage disequilibrium between segregating SNPs [39], [40]. Associations of segregating SNPs with the phenotypic sex of the fish were investigated, fitting a generalized linear model (GLM) with binominal error distribution and logit function in SAS version 9.3. First, a stepwise logistic regression analysis was carried out to detect associations between any of the SNP genotypes coded as 0 (CC), 1 (GC), or 2 (GG) and sex coded as a binary trait (0 = male, 1 = female). Second, a gene substitution model was applied to detect sex specific effects and those of significant SNP driven from the stepwise regression. A stepwise logistic regression revealed a significant effect of SNP *ss831884014* and *ss831884018* on the probability of developing a male functional phenotype (stepwise logistic regression, binary logit, chi-square 9.81, p<0.0017). Both SNPs showed a significant effect of equal magnitude on the male proportion in progenies treated at high temperature as they were in full linkage disequilibrium (*LD*, *r^2^* = 1, no recombination event occurred between the two SNPs). However, SNP *ss831884018* was not further considered as the potentially causal variant due to its position in the non-coding region (intron 6; Table 1). The following model was applied:
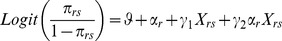
(1)where 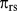 is the probability of obtaining the male phenotype, φ is the overall mean effect, 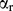 is the fixed effect of the treatment (levels: 28°C or 36°C); 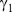 is the linear regression coefficient for association between the probability of obtaining the male phenotype and SNP *ss831884014* genotype, *X_rs_* is the effect of SNP *ss831884014* genotype (levels: 0 (C/C), 1 (G/C), 2 (G/G)); 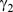 is the linear regression coefficient for the fixed interaction effect. For derivation of the gene substitution effect only the significant parameters were considered in the final model. Since the inverse link is nonlinear and does not give an equal substitution effect, in addition the model parameters were estimated by a general linear model (GLM) using a normally distributed response variable with an identity link function.

**Table 1:** Functional annotation of six allelic variants in the Nile tilapia *amh* gene.

SNP ID	Allele^1^	Consequence^1^	Position	Position in cDNA	Position in CDS	Position in protein	Amino acid change^1^	Codon change^1^
*ss831884015*	C/T	upstream gene variant	5′UTR	-	-	-	-	-
*ss831884016*	A/T	intron variant	Intron 4	-	-	-	-	-
*ss831884017*	C/G	synonymous variant	Exon 5	696	696	232	G (no change)	ggC/ggG
*ss831884014*	G/C	missense variant	Exon 6	1126	1126	376	E/Q	Gaa/Caa
*ss831884018*	G/A	intron variant	Intron 6	-	-	-	-	-
*ss831884019*	T/C	missense variant	Exon 7	1157	1157	386	A/V	gCg/gTg

(^1^The annotation of functional effects was carried out using *SNP*Eff 2.0.5 (http://SNPeff.sourceforge.net/) using the *Oreochromis niloticus* genome sequence deposited in the Ensembl database (scaffold *GL831234.1*)).

Additive and dominance effects of alleles were calculated according to the following model:
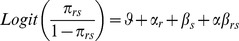
(2)where 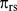 is the probability of obtaining the male phenotype, φ is the overall mean effect, 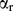 is the fixed effect of the treatment (levels: 28°C or 36°C from 10–20 dpf); 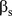 is the fixed effect of SNP *ss831884014* genotype (levels: 0 (C/C), 1 (G/C), 2 (G/G)); 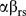 is the fixed effect of interaction between genotype and treatment. First, least square means were estimated on the logit scale and then back-transformed using the inverse link function 
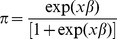
 to the original scale (probability) applying the least square means (LSmeans) statement. Significant differences between least square means were tested using a t-test by inclusion of the PDIFF option in the LSmeans statement. Standard errors of least square means were calculated as described by [41]. Significant deviation of estimates of dominance effects from zero was tested using a t-test as described in [42].

## Results

### Phenotypes: Sex ratios in controls and groups treated at high temperature

Temperature had a significant effect on the sex ratio of the genetically female (XX) line of Nile tilapia consisting of 12 families investigated in the present study. The overall male percentage when reared at control temperature of 28°C, was 2.3%. Losses from 10 dpf until sexing in control groups were 8.6%. In the corresponding full sib groups treated at high temperature, the male percentage was 40.4%, while mortalities were 4.4%.

In the genotyped sample, which was derived from the before mentioned genetically female (XX) line of Nile tilapia consisting of 12 families, a male proportion of 20% in controls, and 53% in the group treated at high temperature was observed. Similarly, the temperature treatment significantly affected the proportion of phenotypic males (p<0.0001).

### Functional annotation of *amh* variants

In total, sequencing of the *amh* gene revealed six SNPs (*ss831884014* to *ss831884019*, Table 1, Figure 2). SNP *ss831884015* (C>T) was detected in the 5′ upstream region, located −225 bp upstream of the start codon, and was classified as intergenic modifier. Two of the identified SNPs were located in introns (intron 4 SNP *ss831884016*, and intron 6 SNP *ss831884018*). Finally, three variants were assigned to exons 5 (*ss831884017*), 6 (*ss831884014*) and 7 (*ss831884019*). SNP *ss831884017* was a synonymous mutation at position 696 of the coding sequence, corresponding to position 232 of the protein. Moreover, SNP *ss831884014* (G>C) and *ss831884019* (C>T) were missense mutations leading to amino acid changes from glutamine to glutamic acid (codons Gaa/Caa) and alanine to valine (codons gCg/gTg).

### Frequency distribution of geno- and haplotypes

Four out of six variants (SNP *ss831884015*, *ss831884014*, *ss831884018*, and *ss831884019*) segregated in the investigated 93 individuals from the genetically female Nile tilapia population (Table 2). Moreover, four phased haplotypes from parent to offspring were reconstructed (C-C-A-C, C-G-G-C, C-G-G-T, T-G-G-T). Variants *ss831884014* and *ss831884018* were in full linkage disequilibrium (*LD*). SNPs *ss831884015* and *ss831884019* also exhibited a high degree of LD, showing an *r^2^*-value of 0.7. The *LD* between SNPs *ss831884014* and *ss831884019* as well as between *ss831884018* and *ss831884019* was intermediate, showing an *r^2^*-value of 0.54. In contrast, *LD* between SNPs *ss831884015* and *ss831884014*, as well as *ss831884015* and *ss831884018* was lower, showing an *r^2^*-value 0.21 (Figure 2, Table S4 in File S1).

**Table 2:** Allele frequencies, polymorphism information content (PIC), heterozygosity and allelic diversity for four allelic variants in the *amh* gene detected in a genetically all-female (XX) Nile tilapia population.

SNP locus	n individuals	Allele frequency	PIC	Heterozygosity	Allelic diversity
*ss831884015*	93	0.90 (C)	0.1595	0.1935	0.1748
*ss831884014*	93^1^	0.71 (C)	0.3245	0.4194	0.4075
	430^2^	0.58 (C)	0.3677	0.5186	0.4856
*ss831884018*	93	0.71 (A)	0.3245	0.4194	0.4075
*ss831884019*	93	0.84 (C)	0.2340	0.3226	0.2706

(^1^ Initially 93 individuals were Sanger-sequenced for the *amh* gene. The genotyped sample was comprised of three families including the corresponding three dams and sires; ^2^ In total 337 individuals were investigated using the FRET-assay, in order to obtain a total of 430 genotypes at SNP *ss831884014* (including the before mentioned Sanger-sequenced individuals)).

### Association of *amh* variant *ss831884014* with autosomal and temperature-dependent sex reversal

A fluorescence resonance energy transfer (FRET) based genotyping assay was developed for SNP *ss831884014* and validated on a representative sample of fish, comprised of 125 individuals reared at control temperature (28°C) and 305 individuals reared at 36°C from 10–20 dpf. The statistical analysis revealed a highly significant effect of the treatment and genotype on the phenotypic sex (Table 3). In contrast, no significant genotype×treatment interaction, and thus no difference in association between fish reared at control or high temperature, was detected (interaction term: df = 424, F = 1.83, p = 0.1618, Table 3). Furthermore, using a reduced model including only the two main factors SNP and treatment as well as applying a Bonferroni correction resulted in significant differences between the group treated at high temperature and the control (p<0.0001), and SNP-genotypes (CC vs. GG; p<0.0007; GC vs. GG; p<0.0067), as no significant interaction between the SNP and treatment was observed (Table 3).

**Table 3:** Effect of SNP *ss831884014* genotypes, temperature treatment (28°C vs. 36°C from 10–20 dpf) and their interaction on the phenotypic sex in Nile tilapia.

Scale	Effect	Equation 1			Equation 2		
		df Numerator	F-statistics	Pr>F	df Numerator	F-statistics	Pr>F
Logit link	treatment	1	4.60	<.0326	1	15.49	<.0001
function	SNP genotype	1	12.66	0.0004	2	5.33	0.0025
	treat*SNP genotype	1	2.63	0.1059	2	1.83	0.1618
Identity link	treatment	1	8.18	<.0044	1	24.02	<.0001
function	SNP genotype	1	12.15	0.0005	2	6.87	0.0012
	treat*SNP genotype	1	0.68	0.4102	2	1.35	0.2593

(The effects were estimated using a GLM with binominal error distribution and logit link function and were validated using a GLM with identity link function; Equation 1: Linear regression model 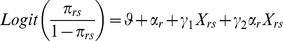; Equation 2: Fixed effect model 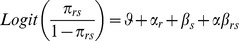.

Generally, the genotype CC showed a more pronounced effect on the percentage of males. Thus genotypic values over both treatments, given as LSmeans, were 0.4224 for the CC- compared to a significantly lower value of 0.1699 for the GG-genotype (p<0.0007). The genotypic LSmean of the heterozygous GC-genotype was 0.3604, which differed significantly from the genotype GG (p<0.0067).

**Figure 3:**
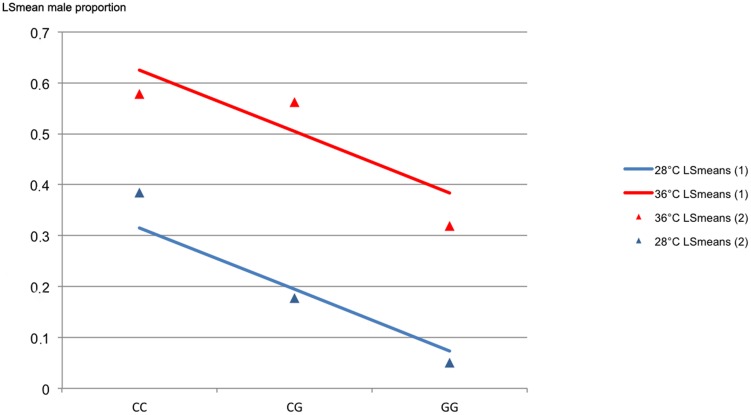
Relationship between male proportion and genotype at *amh* variant *ss831884014* in Nile tilapia, reared at 28°C or 36°C from 10 to 20 days post fertilisation. Linear least-square regression of the genotypic values (LSmean male proportion) for genotypes C/C, G/C, and G/G at locus *ss831884014* of the *amh* gene (Scaffold *GL831234.1*). The genotypic values were derived from the regression models by either excluding the interaction between SNP and treatment (LSmeans 1: red and blue line) or by including the interaction between SNP and treatment (LSmeans 2: red and blue triangles) as effect class. Numbers of individuals per genotype were 26/79/20 and 114/144/47 for genotypes C/C, G/C, and G/G, for the control group (28°C) and the group reared at high temperature (36°C), respectively.

Although no significant interaction between treatments was detected, the genotypic LSmeans derived for both the group treated at high temperature and the control group are given separately in figure 3. The CC-genotype exhibited the highest and the GG-genotype the lowest genotypic LSmean male proportion, irrespective of the treatment. Thus, homozygous CC individuals showed an LSmean proportion of males (57.8%) when treated at high temperature. In comparison, homozygous CC specimens kept at 28°C exhibited LSmeans of 38.4%. The LSmean male proportions dropped to 31.9% and 5.0% for individuals possessing the homozygous genotype GG, in the group treated at high temperature and the control group, respectively.

### Gene substitution effect, homozygous additive allele effect, and dominance effect of *amh* variant *ss831884014* for autosomal and temperature-dependent sex reversal

An analysis of covariance was carried out to estimate the gene substitution effect, where the effect of the SNP-genotype was considered as the regression term (equation 2). The estimated gene substitution effect was 12% for both treatments. Genotypic values derived from the corresponding regression model were 63%, 50%, and 38% for genotypes CC, GC, and GG in fish treated at high temperature. In contrast to this, lower genotypic values of 32%, 19%, and 7% were observed in fish kept at the control temperature of 28°C for genotypes CC, GC, and GG, respectively. Moreover, the genotype effect of SNP *ss831884014* on the male proportion was significantly higher in groups treated at high temperature, yielding 31% higher male ratios (LSmeans) on average of the three genotypes (Figure 3). Furthermore, the homozygous additive effect of the C-allele was estimated, which deals with the increase in male percentage contributed by each copy of the C-allele. This effect was calculated as the mean between the two homozygous genotypes (*a* = μ_CC_−μ_GG_)/2). In controls the homozygous additive allele effect showed a value of 16.7% (S.E. = 6.9), whereas a value of 12.9% (S.E. = 4.04) was observed in fish treated at high temperature (Table 4). In addition to the additive effects of alleles, dominance is of theoretical and practical importance (i.e. in breeding). A significant dominance deviation of 11.4% (S.E. = 5.6) was estimated for the fish treated at high temperature. In contrast, no significant dominance deviation was detected in individuals reared at control temperature (−4.01%, S.E. = 8.6).

**Table 4:** Dominance and additive effects of the variant *ss831884014* on the male proportion in Nile tilapia reared at 28°C or 36°C from 10–20 dpf.

Genetic parameter		Treatment	Estimate^1^	Back transformed Lsmeans^2^	Estimate^3^	t-value^1^	p-Value^1^
Dominance	= μ_CG_−(μ_CC_+μ_GG_)/2	28°C	0.1719 (0.6250)	−0.0400	−0.0400 (0.0868)	0.28	0.7834
	= μ_CG_−(μ_CC_+μ_GG_)/2	36°C	0.4709 (0.2484)	0.1135	0.1135 (0.0560)	1.90	0.0586
Homozygous additive allele effect	= (μ_CC_−μ_GG_)/2	28°C	1.2372 (0.5512)	0.1673	0.1673 (0.0693)	2.24	0.0253
	= (μ_CC_−μ_GG_)/2	36°C	0.5381 (0.1830)	0.1299	0.1299 (0.0404)	2.94	0.0035

(Values given brackets are standard errors of the estimates; ^1)^ Parameters were derived from GLM with binominal error distribution and logit link function; ^2)^ Dominance and homozygous additive allele effects were derived from back transformed LSmeans; ^3)^ Parameters were derived from GLM with identity link function).

## Discussion

Tilapias have the potential to become the most important aquaculture species group world-wide [43]. They serve as a means for poverty alleviation and concurrently constitute a world-wide commodity, being sold as frozen fillets to export markets in the US, Europe, and Asia [44]. More than 50% of seafood consumed comes from aquaculture; tilapia is a major mainstay of this production.

Tilapia differ from other aquaculture species insofar as they can thrive under poor conditions and on diets that contain low shares of fish meal [2], but a major sustainability constraint - i.e. hormonal sex-reversal - is yet to be overcome. The present study reports novel allelic variants in the Nile tilapia *amh* gene. One of the variants is associated with the sex and might enable marker-assisted selection. A strategy is proposed to increase the male proportion by the development of a straightforward genotyping assay combined with temperature treatment of fry.

### Association of *amh* variant *ss831884014* with autosomal and temperature-dependent sex reversal

The largest effect on the phenotypic sex of Nile tilapia was observed in homozygous carriers of the C-allele at SNP *ss831884014*, a missense variant that leads to an amino acid change from glutamine to glutamic acid at position 376 of the putative *amh* protein. Moreover, a ten-day temperature treatment of the sexually undifferentiated fry carrying the homozygous CC genotype significantly increased the probability of developing the male phenotype. Although the homozygous effect of the C-allele was higher in the control groups (16.7% compared to 12.9% males), the average genotypic values were 31% higher (LSmean male ratios) in groups treated at high temperature.

Two factors were identified which suggest that the C-allele (in homozygous and heterozygous allelic state) in our population has the potential to significantly increase the proportion of males: 1) Genotypic values of 63% and 32% males for the CC-genotype and 38% and 7% for the alternative GG-genotype in the group treated at high temperature and control group, respectively; 2) A significant dominance deviation (+11.4% of males) of the heterozygous (CG) genotype in the fish treated at high temperature.

### Comparative genomics and functional role of the *amh* variant *ss831884014*

A comparative analysis of the *amh* protein revealed that the G-allele might be the ancestral allele at locus *ss831884014* (Figure 2, panel e). Species such as tilapia, stickleback, and medaka have the amino acid glutamic acid (E: coded by Gaa, Gag) in their protein sequence, whereas puffer fish and platy fish exhibit the acidic amino acid aspartate (D: coded by Gac, Gat). Hence, this might indicate that glutamine (Q), which is coded by the nucleotide codon Caa, is more recent in the Nile tilapia line investigated here and might have taken over a critical role in autosomal and temperature-dependent sex reversal.

The *amh* gene was identified as a potentially sex-modifying cue in a number of earlier studies [15], [16], [18], [22], [32]–[34]. On the one hand, expression analysis confirmed *amh* as a functional marker of maleness at a precocious age of 3 dpf and later during sex differentiation [15], [32]–[34]. On the other hand, evidence for sex-specific differences in the *amh* gene at the genomic level was lacking so far. An earlier identified variant (*AM232733*), similarly located in exon 6 of the gene, proved not to be a trigger for sexual fate in Nile tilapia [18]. Moreover, the Nile tilapia population investigated here (Lake Manzala population), showed no segregation of variant *AM232733*. However, among 10 genes putatively involved in sex determination or differentiation, *amh* is located in the centre of an SD QTL on LG23 [15].

### The role of the *amh* variant *ss831884014* in the continuum of major genetic, minor genetic, and temperature-dependent factors

The present study does confirm - for the first time - that an allelic variant in the *amh* gene might be a major QTL for autosomal and temperature-dependent sex reversal in Nile tilapia. In the Patagonian pejerrey a functional duplication of the *amh* gene suggests that *amhy* may be the master sex-determining gene [45], but our results for tilapia point in another direction: multiple interacting loci each partially contributing to the formation of the sexual phenotype. Although the allelic variant reported here exerts a large effect on the formation of the sexual phenotype under both rearing environments, not all sex phenotypes can be explained using a simple one-locus model. Recently, restriction site associated sequencing revealed the main sex-determining locus flanked by two SNPs (*Oni23063* and *Oni28137*) and located on linkage group 1 [46]. The authors showed that in one family, sex-reversed males which were treated at high temperature carried the female genotype at SNP *Oni23063* and *Oni28137*, but did not show an association with the temperature-dependent sex [46]. The underlying gene of the main sex-determining locus on LG1 is unknown so far. Further studies are needed to verify if SNP *ss831884014* on LG23 acts as autosomal sex modifying cue in this Nile tilapia line, which in addition or alternatively to the major genetic factor on LG1, described in [46], increases the probability of developing the male phenotype during temperature-dependent sex reversal. The missing significant interaction between the SNP-genotype and treatment effect detected in the present study (Table 3) suggests that the same genetic network (i.e. the *TGF-ß* pathway) might be at least partially active during autosomal and temperature-dependent sex reversal in this Nile tilapia line, yet with a drastically pronounced effect under elevated rearing temperatures from 10–20 dpf.

Further research should now focus on the functional role of the identified allelic *amh* variant during autosomal and temperature-dependent sex reversal. The underlying gene networks, such as the *TGF-ß* pathway, need to be investigated in greater depth, and their transcriptional, translational, and phenotypic consequences have to be evaluated to fully characterize the effect on the phenotypic sex described here. Targeted techniques such as RNA-seq [47], or morpholino-mediated knockdown of known target genes might reveal the functional role of genes and variants [48]. Another avenue of enquiry focuses upon the specific biochemical properties of the *amh* hormone. The combined use of temperature treatment and marker-assisted selection to introgress the C-allele at SNP *ss831884014* into other strains of cultivated tilapia might help to increase the male proportion and therefore the overall productivity. Ultimately, increasing the yield through higher male proportions without counteracting reproductive traits would greatly contribute to increase the efficiency of Nile tilapia culture and protein availability in predominant small-scale aquaculture, while also minimizing negative environmental effects due to hormonal sex-reversal from expanding intensive aquaculture systems.

### Conclusions

This study shows that an allelic variant in the *amh* gene is a major QTL for autosomal and temperature-dependent sex reversal in a selected line of Nile tilapia. If the applicability of this marker in other populations proofs successful, a combined strategy of marker-assisted selection and temperature treatment might be beneficial to increase the male proportion in aquaculture stocks of *O.niloticus*.

## Supporting Information

File S1 **Contains supporting Figures and Tables.** Figure S1, Melt curve analysis output from the Lightcycler 480 system for genotypes at *amh* variant *ss831884014*. Different genotypes are illustrated using different colours: C/C = green curve, C/G and G/C = blue curve, and G/G = red curve. Homozygeous C/C individuals show a fluorescence peak between 483 and 610 nm wave length at 58°C, whereas homozygeous G/G individuals showed one at 64°C, and heterozygous fish showed two peaks. Table S1, Pedigree and sex ratios of the genetically female population reared at control (28°C) and elevated temperature (36°C) from 10 to 20 dpf. Table S2, Forward and reverse primers tailed with a universal M13 forward or reverse primer for bidirectional sequencing the *amh* gene in Nile tilapia. Table S3, Fret-primer for allelic variant 1690582 in the Nile tilapia *amh* gene, anchor and sensor probe sequences and positions on scaffold *GL831234.1.* Table S4, R^2^-measure of linkage disequilibrium between four segregating allelic variants in the *amh* gene of Nile Tilapia. The estimates were derived from a sample 93 temperature-treated Nile tilapia individuals. Table S5, Raw data for the genetically female study population reared at control (28°C) and elevated temperature (36°C) from 10 to 20 dpf. Table S6, Genotypes of four segregating SNPs in the *amh* gene of 93 individuals derived from three Nile tilapia families.
(ZIP)
