# Correction: Allelic Variant in the Anti-Müllerian Hormone Gene Leads to Autosomal and Temperature-Dependent Sex Reversal in a Selected Nile Tilapia Line

**DOI:** 10.1371/journal.pone.0114341

**Published:** 2014-11-25

**Authors:** The PLOS ONE Staff

Sabrina Pach should be listed in the Acknowledgments rather than the Author Byline. The correct Author Byline is:

Stephan Wessels^1*^, Reza Ahmad Sharifi^2^, Liane Magdalena Luehmann^1^, Sawichaya Rueangsri^3^, Ina Krause^1^, Gabriele Hoerstgen-Schwark^1^, Christoph Knorr^4^

1 Department of Animal Sciences - Aquaculture and Water Ecology, Goettingen University, Goettingen, Germany,

2 Department of Animal Sciences - Animal Breeding and Genetics, Goettingen University, Goettingen, Germany,

3 Department of Animal and Aquatic Sciences, Faculty of Agriculture, Chiang Mai University, Chiang Mai, Thailand,

4 Department of Animal Sciences - Livestock Biotechnology and Reproduction, Goettingen University, Goettingen, Germany

* Email: swessel@gwdg.de

The correct Acknowledgments statement is: We thank Birgit Reinelt for her technical assistance in the recirculation system and help during sampling of fish. We are grateful to E. Schütz for designing the FRET-probes. We thank Sabrina Pach for her technical support during the laboratory work.

Two references are omitted from the third sentence under the subheading “Genotyping of SNP *ss831884014* within *amh*” of the Materials and Methods section. The correct sentence should read: Primers Fret-for and Fret-rev were designed to flank SNP *ss831884014* yielding a fragment of 122 bp length (Schütz et al,1999, Von Ahsen et al, 1999).

The references are:

Schütz E, von Ahsen N (1999) Spreadsheet software for thermodynamic melting point prediction of oligonucleotide hybridization with and without mismatches. Biotechniques 27: 1218–1224.

Von Ahsen N, Oellerich M, Armstrong VW, Schütz E (1999) Application of a thermodynamic nearest-neighbor model to estimate nucleic acid stability and optimize probe design: prediction of melting points of multiple mutations of apolipoprotein B-3500 and factor V with a hybridization probe genotyping assay on the LightCycler. Clin Chem 45: 2094–2101.
