# Supplementary material for: Allelic Variant in the Anti-Müllerian Hormone Gene Leads to Autosomal and Temperature-Dependent Sex Reversal in a Selected Nile Tilapia Line
Source: PLoS One. 2014 Aug 26;9(8):e104795. doi: 10.1371/journal.pone.0104795 (PMC4144872; doi:10.1371/journal.pone.0104795)
Supplement: File S1 — Contains supporting Figures and Tables. Figure S1, Melt curve analysis output from the Lightcycler 480 system for genotypes at amh variant ss831884014. Different genotypes are illustrated using different colours: C/C = green curve, C/G and G/C = blue curve, and G/G = red curve. Homozygeous C/C individuals show a fluorescence peak between 483 and 610 nm wave length at 58°C, whereas homozygeous G/G individuals showed one at 64°C, and heterozygous fish showed two peaks. Table S1, Pedigree and sex ratios of the genetically female population reared at control (28°C) and elevated temperature (36°C) from 10 to 20 dpf. Table S2, Forward and reverse primers tailed with a universal M13 forward or reverse primer for bidirectional sequencing the amh gene in Nile tilapia. Table S3, Fret-primer for allelic variant 1690582 in the Nile tilapia amh gene, anchor and sensor probe sequences and positions on scaffold GL831234.1. Table S4, R2-measure of linkage disequilibrium between four segregating allelic variants in the amh gene of Nile Tilapia. The estimates were derived from a sample 93 temperature-treated Nile tilapia individuals. Table S5, Raw data for the genetically female study population reared at control (28°C) and elevated temperature (36°C) from 10 to 20 dpf. Table S6, Genotypes of four segregating SNPs in the amh gene of 93 individuals derived from three Nile tilapia families. (ZIP) [file pone.0104795.s001.zip › Supplementary Table 2.docx]

**Allelic variant in the anti-Müllerian hormone gene leads to phenotypic sex reversal In Nile tilapia**

Table S2. Forward and reverse primers tailed with a universal M13 forward or reverse primer for bidirectional sequencing the *amh* gene in Nile tilapia.

| Primer name | Position | Start position on scaffold *GL831234.1* | Stop position on scaffold *GL831234.1* | Primer sequence (5'→3') |
| --- | --- | --- | --- | --- |
| Forward 0 | 5’ UTR | 1688299 | 1688317 | TGTAAAACGACGGCCAGTCGAGAGGGTTAAGTTAAGCAGC |
| Forward 0 | Exon 2 | 1688925 | 1688944 | CAGGAAACAGCTATGACCAAGCTGCTGCTGAGGATGATGA |
| Forward 1 | Exon 1 | 1688687 | 1688706 | TGTAAAACGACGGCCAGTATGTTGGGTCTGCTCGTTCT |
| Reverse 1 | Exon 3 | 1689284 | 1689303 | CAGGAAACAGCTATGACCAAGAGCAGCACAGGGTTTGT |
| Forward 2 | Exon 2 | 1688687 | 1688706 | TGTAAAACGACGGCCAGTACTCACTCCCATCAGCGAAG |
| Reverse 2 | Intron 3 | 1689571 | 1689593 | CAGGAAACAGCTATGACCAAACAAGTGCAATTTGTACACCA |
| Forward 3 | Intron 3 | 1689609 | 1689631 | TGTAAAACGACGGCCAGTGATGTCTCAGTGATGAAATGTGC |
| Reverse 3 | Exon 6 | 1690161 | 1690180 | CAGGAAACAGCTATGACCAAGCACATCTGGGAAAGCAA |
| Forward 4 | Exon 6 | 1689803 | 1689822 | TGTAAAACGACGGCCAGTGCTGTGTGCATTTCAGGAGA |
| Reverse 4 | Exon 6 | 1690357 | 1690376 | CAGGAAACAGCTATGACCGTGGGAGCTGTGGAGTTGAT |
| Forward 5 | Exon 6 | 1690374 | 1690393 | TGTAAAACGACGGCCAGTCACAGTCTTTGGCTTCACGA |
| Reverse 5 | Intron 6 | 1691041 | 1691060 | CAGGAAACAGCTATGACCCAGCCAAGCTCACACACACT |
| Forward 6 | Intron 6 | 1690842 | 1690861 | TGTAAAACGACGGCCAGTCGGTCCCAGTGACCTATGAG |
| Reverse 6 | Exon 7 | 1691732 | 1691751 | CAGGAAACAGCTATGACCGCATCTGGCTTGATGGAGAT |
